# Synthesis, Characterization, X-ray Structure and Biological Activities of C-5-Bromo-2-hydroxyphenylcalix[4]-2-methyl resorcinarene

**DOI:** 10.3390/molecules181113369

**Published:** 2013-10-29

**Authors:** Hamza M. Abosadiya, Siti Aishah Hasbullah, Mukram Mohamed Mackeen, Seow Chew Low, Nazlina Ibrahim, Mamoru Koketsu, Bohari M. Yamin

**Affiliations:** 1 School of Chemical Sciences and Food Technology, Universiti Kebangsaan Malaysia, Bangi 43600, Selangor, Malaysia; E-Mails: hamza_inorg@yahoo.com; (H.M.A.); aishah80@ukm.my (S.A.H.); mukram@ukm.my (M.M.M.); 2 Institute of Systems Biology (INBIOSIS), Universiti Kebangsaan Malaysia, Bangi 43600, Selangor, Malaysia; 3 School of Biosciences and Biotechnology, Universiti Kebangsaan Malaysia, Bangi 43600, Selangor, Malaysia; E-Mails: alicia.w05@gmail.com (S.C.L.); nazlina@ukm.my (N.I.); 4 Department of Chemistry and Biomolecular Sciences, Gifu University, 1-1 Yanagido, Gifu 501-1193, Japan; E-Mail: koketsu@gifu-u.ac.jp

**Keywords:** C-5-bromo-2-hydroxyphenylcalix[4]-2-methylresorcinarene, biological activities, X-ray structural study

## Abstract

C-5-bromo-2-hydroxyphenylcalix[4]-2-methylresorcinarene (**I**) was synthesized by cyclocondensation of 5-bromo-2-hydroxybenzaldehyde and 2-methylresorcinol in the presence of concentrated HCl. Compound **I** was characterized by infrared and nuclear magnetic resonance spectroscopic data. X-ray analysis showed that this compound crystallized in a triclinic system with space group of Pī, a = 15.9592(16)Å, b = 16.9417(17)Å, c = 17.0974(17)Å, α = 68.656(3)°, β = 85.689(3)°, γ = 81.631(3)°, Z = 2 and V = 4258.6(7)Å^3^. The molecule adopts a chair (C_2h_) conformation. The thermal properties and antioxidant activity were also investigated. It was strongly antiviral against HSV-1 and weakly antibacterial against Gram-positive bacteria. Cytotoxicity testing on Vero cells showed that it is non-toxic, with a CC_50_ of more than 0.4 mg/mL.

## 1. Introduction

Calixarenes are now important technology materials. Their unique size and cup structure cavities can accommodate some ions and neutral molecules via host-guest interaction and have driven supramolecular chemistry applications in many fields [1,2,3,4,5]. Calixarenes have been used as additives in capillary electrophoresis, liquid membranes, extraction process, chemical sensing and HPLC stationary phases. A number of calix[4]resorcinarene compounds have shown a potential as adsorbents for heavy metal separation [6,7]. Among all known resorcinarenes, calix[4]resorcinarenes are the most widely investigated group of compounds. Basically, they can be synthesized via the acid-catalyzed condensation reaction of resorcinol with aldehydes [8]. One of the important properties of resorcinarenes is their ability to bind strongly with solvent molecules that has great influence on their stability. TGA studies have shown that the solvate molecules such as alcohol, pyridine and DMF were removed at about 300 °C [9]. Therefore, most of the crystal structures of calix[4]resorcinarenes reported so far have 1 to 3 or more solvated molecules. Many of the calix[4]resorcinarenes that we have attempted to crystallize changed their crystalline forms into whitish solids or powders after standing for few hours or even minutes at room temperature. In fact for some compounds, instability was observed while in solution. There is interest in investigating in more detail the flexibility among the possible conformers (crown, boat, chair, diamond and saddle) in the crystalline state and the role of solvents. In the present study, C-5-bromo-2-hydroxyphenylcalix[4]-2-methylresorcinarene (**I**) was synthesized and recrystallized from DMF. The crystal was quickly coated with the Princeton-H oil and mounted on a glass fiber and the X-ray experiment was conducted at low temperature. After a number of experiments, a reasonably good data set was obtained. This article describes the synthesis, characterization, crystal structure, thermal and biological properties of C-5-bromo-2-hydroxyphenyl-calix[4]-2-methylresocinarene (**I**).

## 2. Results and Discussion

### 2.1. Synthesis and Characterization

**Scheme 1:**
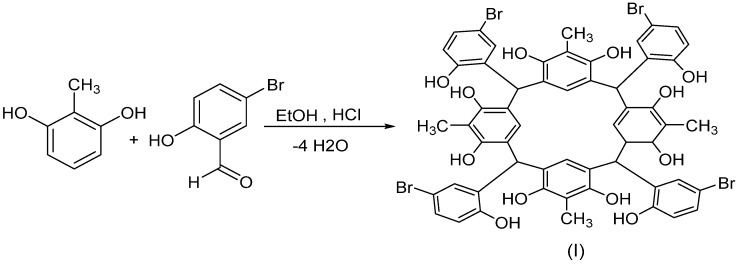
Synthesis of C-5-bromo-2-hydroxyphenylcalix[4]-2-methylresorcinarene (**I**).

The synthesis of C-5-bromo-2-hydroxyphenylcalix[4]-2-methylresorcinarene (**I**) was accomplished by refluxing mixture of equal molar amounts of 2-methylresorcinol with 5-bromo-2-hydroxybenzaldehyde in the presence of concentrated HCl in EtOH at 80 °C (Scheme 1).

**Figure 1:**
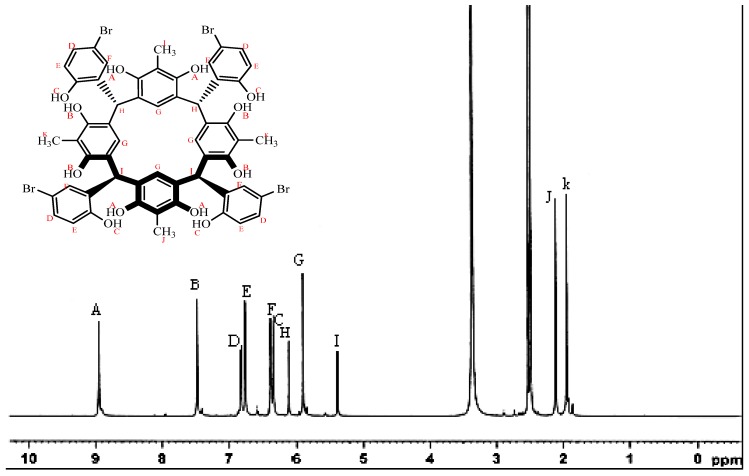
^1^H-NMR spectrum of C-5-bromo-2-hydroxyphenylcalix[4]-2-methylresorcinarene in DMSO-*d*_6_.

The ^1^H-NMR spectrum of compound **I** (Figure 1) showed a clean two sets of phenolic protons at 7.48 and 8.94 ppm, respectively, belonging to the resorcinarene rings. The phenolic proton of the *p*-bromophenyl ring appeared upfield at 6.38 ppm. The four methyl groups of the resocinarene also appeared in two sets of signals at 1.94 and 2.11 ppm, respectively. It is known that the phenolic protons of the resorcinarene and methine protons are sensitive to the chemical environment of the different conformations of the resorcinarene [10,11,12]. Although mixed conformations are quite common in solution, for the present compound, the two sets of the hydroxyl protons of resorcinol (7.48 and 8.94 ppm) and the methine protons (5.37 and 6.10 ppm) indicate a high degree of co-planarity between the resorcinol rings and lead to the adoption of a chair conformation. The ^13^C-NMR spectrum showed the phenolic carbon of the bromohydroxybenzene fragment at 153.8 ppm whereas the two sets of resorcinol carbons appeared at 150.9 and 151.2 ppm. The corresponding two sets of methyl carbons are at 10.4 and 10.6 ppm, respectively. The four tertiary methine carbons appeared at 36.8 ppm. The ^1^H-NMR spectrum of the crystallized calix is very similar to that of the solid precipitate product, except for the presence of extra signals at 2.73, 2.89 and 7.95 ppm, respectively, due to the two methyls and the aldehyde proton of the DMF solvent. 

### 2.2. X-Ray Structure

**Figure 2:**
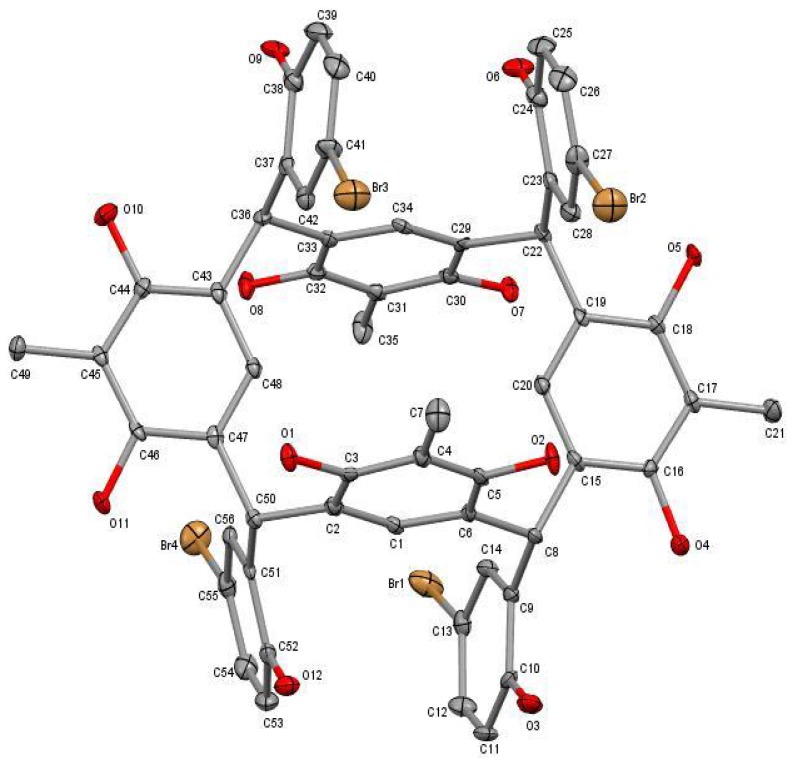
The molecular structure of C-5-bromo-2-hydroxyphenylcalix[4]-2-methyl-resorcinarene drawn at 50% probability ellipsoid. The hydrogen atoms and solvent molecules are not shown for clarity.

The X-ray investigation showed that compound (**I**), crystallized in DMF, possesses a triclinic system with the space group Pī, a = 15.9592(16) Å, b = 16.9417(17) Å, c = 17.0974(17) Å, α = 68.656(3)°, β = 85.689(3)°, γ = 81.631(3)°, Z = 2 and V = 4258.6(7) Å^3^. The asymmetric unit consists of one C-5-bromo-2-hydroxyphenylcalix[4]-2-methylresorcinarene at special position, eight DMF and four water molecules of crystallization. The calix molecule adopts a chair conformation (C_2h_) with two opposite resorcinol groups almost coplanar to each other and a pair of them that are anti-parallel (Figure 2).

The dihedral angle between the opposite resorcinol rings (C15–C20) and (C43–C48) is 5.8(2)° whereas the anti-parallel rings (C1–C6) and (C29–C34) have a dihedral angle of 1.4(2)°. The alternate resorcinol rings (C1–C6) and (C15–C20) are perpendicular with a dihedral angle of 77.2(2)°. The two bromo-hydroxyphenyl linkage groups that are attached to both sides of the co-planar resorcinol rings are in opposite directions, whereas two rings face upwards and the other two rings directly downwards. A similar conformation with approximately C_2h_ symmetry due to the presence of a crystallographic inversion center has also been observed for tetraarylboronic acid resorcinarene [13]. The bond lengths and angles (Table 1) are in normal ranges and comparable to those of other resorcinarenes [14]. The co-crystallized solvent molecules are located outside the calix molecule and therefore the cavity remains empty, which enables it to participate in the host-guest activity.

**Table 1:** Selected bond lengths and angles of C-5-bromo-2-hydroxyphenylcalix[4]-2-methylresorcinarene (**I**).

Bond	Length Å	Bond	Angles °
Br1-C13	1.905(6)	C12-C13-Br1	120.0(4)
Br2-C27	1.902(5)	C26-C27-Br2	120.6(4)
Br3-C41	1.904(6)	C40-C41-Br3	118.8(4)
Br4-C55	1.905(5)	C54-C55-Br4	119.8(4)
O1-C3	1.381(6)	O1-C3-C2	116.9(5)
O2-C5	1.390(6)	O2-C5-C4	117.2(5)
O3-C10	1.359(7)	O3-C10-C11	122.6(5)
O4-C16	1.366(6)	O4-C16-C15	116.0(5)
N1-C59	1.319(8)	C59-N1-C58	120.6(6)
N2-C62	1.314(9)	C62-N2-C60	120.6(6)
N3-C65	1.325(8)	C65-N3-C64	121.5(5)
N4-C68	1.327(8)	C68-N4-C67	120.8(6)

**Figure 3:**
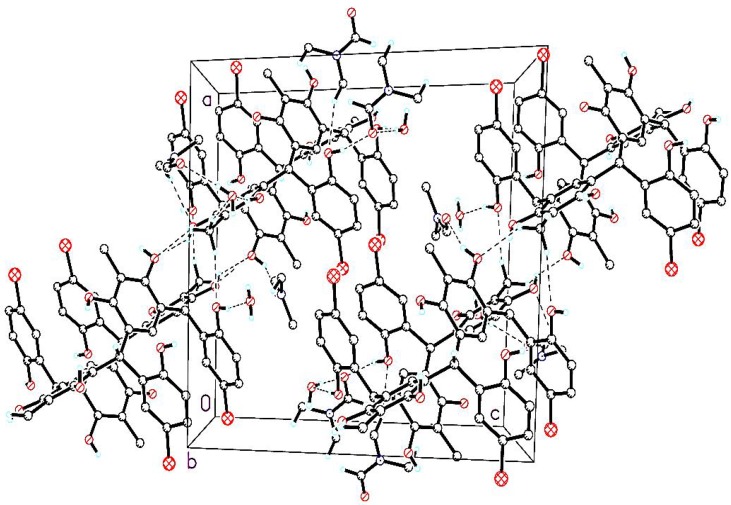
Molecular packing of C-5-bromo-2-hydroxyphenylcalix[4]-2-methylresorcinarene, viewed down the b axis. Dashed lines denote O—H^….^O and C—H^….^O hydrogen bonds and the non-hydrogen bonded atoms are omitted for clarity.

There are no significant intramolecular hydrogen bonds in the calix moiety. In the crystal structure, two calix molecules are connected by C—H^…^O hydrogen bonds between the methyl hydrogen atoms and the resorcinol oxygen atoms to form dimers (Figure 3) which then connected to the water and DMF solvent molecules by O—H^…^O and O^...^H—C hydrogen bonds, respectively (symmetry codes as shown in Table 2).

**Table 2:** Hydrogen geometric parameters (Å) of C-5-bromo-2-hydroxyphenylcalix[4]-2-methylresocinarene.

D―H^….^A	D―H	H^….^A	H^….^A	D―H^….^A
O1W―H1WB^.....^O17	0.82(6)	1.94(6)	2.756(8)	172(3)
O3―H3^….^O20	0.84	1.86	2.690(6)	169
O2W―H2WB^…..^O20	0.82(4)	2.08(4)	2.895(7)	173(6)
O7―H7^….^O15	0.84	1.83	2.576(6)	146
O12―H12^….^O2W	0.84	1.86	2.696(7)	174
C64―H64B^….^O1W	0.98	2.60	3.514(9)	155
C66―H66B^….^O3W	0.98	2.59	3.400(9)	140
C72―H72B^….^O4	0.98	2.36	3.337(9)	172
C76―H76A….O18	0.98	2.59	3.440(8)	145
O1―H1^…..^O19 ^ii^	0.84	2.08	2.821(6)	148
O1W―H1WA^….^O13 ^vii^	0.82(7)	2.03(7)	2.815(7)	161(6)
O2W―H2WA^….^O16 ^ix^	0.82(3)	1.91(4)	2.718(7)	168(10)
O4―H4….O3W ^iii^	0.84	1.9	2.668(6)	152
O5―H5^….^O7 ^iv^	0.84	2.06	2.796(5)	145
O3W―H3WA^….^O13 ^xi^	0.82(5)	1.96(6)	2.756(7)	162(6)
O6―H6^….^O1W ^iv^	0.84	1.89	2.718(7)	170
O3W―H3WB^....^O14 ^x^	0.82(5)	2.13(5)	2.945(7)	174(9)
O4W―H4WA^….^O17 ^viii^	0.82(6)	1.94(6)	2.764(8)	174(7)
O8―H8^….^O18 ^v^	0.84	2.01	2.811(6)	159
O4W―H4WB^....^O14 ^iii^	0.82(5)	1.98(5)	2.778(10)	166(7)
O9―H9^….^O4W ^vi^	0.84	1.87	2.706(7)	170
O10―H10^….^O18 ^v^	0.84	1.91	2.730(6)	165
O11―H11^….^O1 ^i^	0.84	2.05	2.786(5)	147
C21―H21A^….^O3W ^iii^	0.98	2.37	3.325(9)	165
C21―H21B^….^O7 ^iv^	0.98	2.47	3.305(7)	143
C21―H21C^….^O6 ^iv^	0.98	2.53	3.455(8)	158
C36―H36^….^O18 ^v^	0.98	2.38	3.255(7)	146
C49―H49B^….^O1 ^i^	0.98	2.59	3.444(6)	146
C59―H59^….^O4W ^vi^	0.95	2.51	3.441(8)	167
C61―H61B^….^O5 ^iv^	0.98	2.57	3.509(7)	160
C75―H75A^….^O15 ^iii^	0.98	2.53	3.453(8)	157
C78―H78A^….^O16 ^ix^	0.98	2.59	3.331(9)	133
C79―H79C^….^O3 ^ii^	0.98	2.55	3.487(8)	161
C80―H80^….^O16 ^iii^	0.95	2.59	3.531(8)	171

Symmetry codes: i = −x, 2 − y, 1 − z; ii = −x, 1 − y, 1 − z; iii = 1 − x, 1 − y, 1 − z; iv = 1 − x, 1 − y, 2 – z; v = x, 1 + y, z; vi = x, 1 + y, 1 + z; vii = x, −1 + y, z; viii = 1 − x, −y, 1 – z; ix = −1 + x, y, z; x = 1 − x, 1 − y, 2 − z; xi = 1 − x, 2 − y, 1 − z.

### 2.3. Thermogravimetric Study

**Figure 4:**
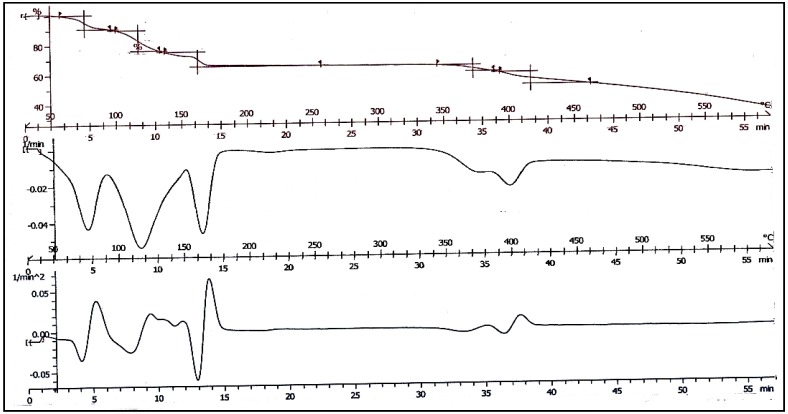
Thermogram of C-5-bromo-2-hydroxyphenylcalix[4]-2-methylresocinarene (**I**).

It has been reported based on TGA studies that the thermal decomposition of resorcinarene can be very complicated due to the presence of large numbers of solvent molecules [15]. In many cases, the solvent molecules were released at higher temperature than their boiling points, indicating strong binding among the molecules in the solid state. It is also possible that solvents such as DMF decompose during the thermal process and the decomposition products react with each other. Such complexity is also depicted by the thermogram of the present calix (Figure 4). At least five mass loss steps occurred at about 80, 120, 165, 370 and 400 °C, respectively. The second derivative plot (DTA) showed the possibility of more than two steps in the second mass loss step. The first mass loss is normally due to the release of water molecules, but the calculated mole percentage of water (3.8%) is very much lower than the experimental loss of 9.62%, indicating the release of a different product or mixture of products. The final residue of decomposition is carbon. A more detailed study on the thermal decomposition of the calix is necessary to further elucidate the multiple mass losses.

### 2.4. Biological Studies

#### 2.4.1. Antioxidant Properties

Antioxidant properties measured as radical scavenging activity are due to the transfer of electrons or hydrogen atoms of the hydroxyl groups to an oxidizing agent. Compound **I**, being a polyphenolic compound, could inhibit the oxidation of other molecules such as 1,1-diphenyl-2-picryl-hydrazyl (DPPH) by donating hydrogen atoms to form the stable non-radical form of DPPH as shown by the formation of a pale yellow color. The antioxidant activity exhibited by compound **I** was 84.95%, which is very close to that of the compound 5,11,17,23,-tetra-*tert*-butyl-25,27-bis(5-(hexanyl)-1,3,4-oxadiazole-2-thiacarbonylmethoxy)-26,28-dihydroxycalix[4]arene [16] indicating high antioxidant capability.

#### 2.4.2. Antibacterial Activity

**Figure 5:**
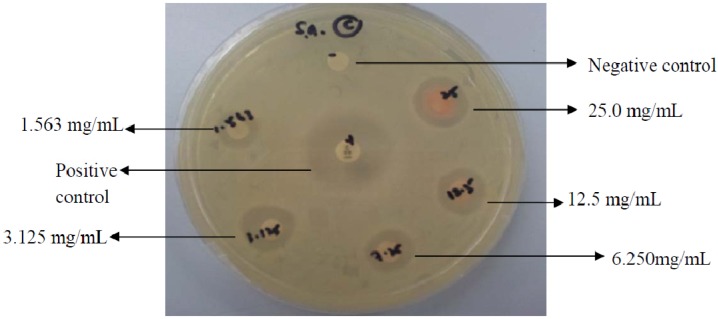
Inhibition zones of C-5-bromo-2-hydroxyphenylcalix[4]2-methylresorcinarene (**I**) against MRSA using the disc diffusion assay tested at concentrations of two-fold dilution. The highest concentration of tested would correspond to 250 μg.

The antibacterial activity of C-5-bromo-2-hydroxyphenylcalix[4]-2-methylresorcinarene (**I**) on five test bacteria (Gram-positive and -negative) is shown in Table 3. Results showed that compound **I** only displayed antibacterial activity against Gram-positive bacteria, namely methicilin-resistant *Staphylococcus aureus* (MRSA) (Figure 5) with inhibition zones between 10 and 15 mm. However, the antibacterial activity was weaker than the antibiotic standards. 

**Table 3:** Diameter of inhibition zone for antibacterial screening of C-5-bromo-2-hydroxy phenylcalix[4]-2-methylresorcinarene (**I**).

Dose (µg)	Diameter of inhibition zone (mm)				
	MRSA	Sa	Ef	Ea	Pa
250	13	13	15	6	6
125	12	12	13	6	6
62.5	12	11	11	6	6
31.25	11	11	11	6	6
15.63	10	10	10	6	6
Antibiotic control (30 µg)	15 ^a^	22 ^b^	23 ^b^	26 ^b^	16 ^b^
DMSO (solvent control)	6	6	6	6	6

Notes: MRSA = methicillin-resistant *Staphylococcus aureus*; Sa = *Staphylococcus aureus*; Ef = *Enterococcus faecalis*; Ea = *Enterobacter aerogenes*; Pa = *Pseudomonas aeruginosa*; a = vancomycin; b = chloramphenicol (30 µg). SD inhibition zone = ± 1 mm (biological replicates, 3).

The minimum inhibitory concentration (MIC) and minimum bactericidal concentration (MBC) of compound **I** were also determined (Table 4). Consistent with the disc diffusion test results, compound **I** only inhibited the Gram-positive bacteria. The MIC values revealed that the calix completely inhibited the growth of the tested Gram-positives between 1.563 to 6.25 mg/mL which would be considered weak. Therefore, it is interesting that the antibacterial activity against MRSA in the disc diffusion assay was comparable to the compared antibiotic standard, but was much weaker in the broth dilution assay, thereby not showing low μg/mL MIC values. MBC/MIC value against MRSA is 16 which indicate a bacteriostatic mode of action as opposed to the MBC/MIC values of 2 for both *Staphylococcus aureus* and *Enterococcus faecalis*, which indicate bactericidal action. MBC to MIC ratios of >4 have been defined as bacteriostatic [17].

**Table 4:** Minimum inhibition concentration (MIC) (mg/mL), minimum bactericidal concentration (MBC) (mg/mL) and selectivity index (SI) of C-5-bromo-2-hydroxophenylcalix[4]-2-methylresorcinarene (**I**).

Microorganism	MIC mg/mL	MBC mg/mL	SI
MRSA (Gram-positive)	1.563	25	0.256
Sa (Gram-positive)	6.25	12.5	0.064
Ef (Gram-positive)	6.25	12.5	0.064
Ea (Gram-negative)	>25	-	-
Pa (Gram-negative)	>25	-	-

Note: MRSA = methicillin-resistant *Staphylococcus aureus*); Sa = *Staphylococcus aureus*); Ef = *Enterococcus faecalis*; Ea = *Enterobacter aerogenes*; Pa = *Pseudomonas aeruginosa* (-ve); SI = selectivity index = CC_50_/MIC (refer to Section 2.4.3).

#### 2.4.3. Cytotoxicity Studies

**Figure 6:**
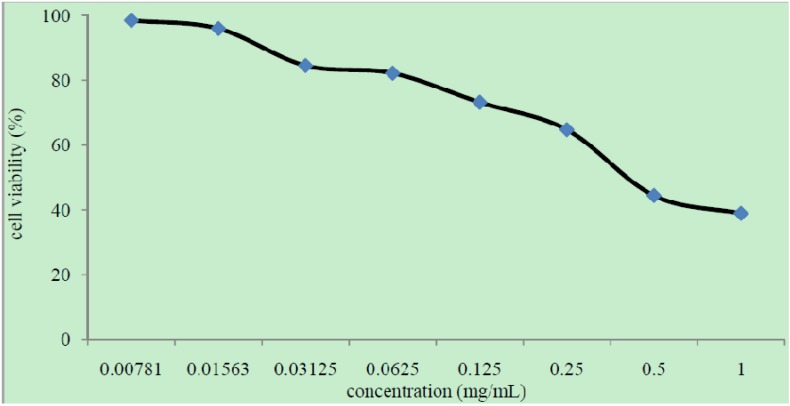
Percentage of cell survival against concentration of compound C-5-bromo-2-hydroxyphenylcalix[4]-2-methylresorcinarene (**I**).

The cytotoxicity test indicated that C-5-bromo-2-hydroxyphenylcalix[4]-2-methylresorcinarene (**I**) is safe to be used as an antimicrobial therapeutic agent due to its non-toxicity towards Vero cells with a CC_50_ value of 0.4 mg/mL. According to Zirihi *et al.* [18], a test compound is considered toxic if the CC_50_ value is less than 0.02 mg/mL. CC_50_ value can be obtained directly from the graph of percentage of cell survival viability versus compound concentration (Figure 6). However, when the CC_50_ value was used to calculate the selectivity index (SI) of antibacterial activity using the equation, SI = CC_50_/MIC, the SI values were lower than 1. Although the CC_50_ value is high indicating non-cytotoxicity, the high MIC value shows weak antibacterial activity. This resulted in a low SI value and suggests that compound **I** is unsuitable as a potential antibacterial agent [19].

#### 2.4.4. Antiviral Activity towards HSV-1

**Figure 7:**
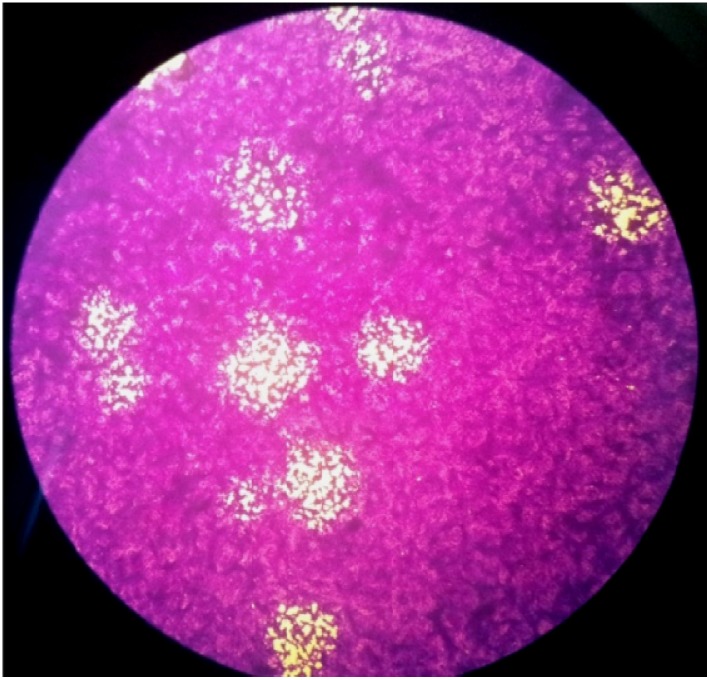
Plaque formation to determine virus titer.

Antiviral tests showed that the compound **I** was suitable as an antiviral agent because of its ability to inhibit 100% plaque formation, even at the lowest concentration of 0.011 mg/mL (Figure 7). Thus, the EC_50_, which is the concentration when the presence of test compound caused 50% reduction of plaques or cytopathic effect, is much lower than the minimum inhibitory concentration of 0.011 mg/mL. The selectivity index (SI = CC_50_/EC_50_) of compound **I** is more than 36. This indicates that compound **I** can be considered as a potentially safe antiviral agent with low cytotoxicity and high potency. SI values greater than 10 indicate potential antiviral therapeutic safety and efficacy.

## 3. Experimental

### 3.1. Materials and Physical Measurements

All the compounds utilized in this work were commercially available high purity products purchased from Acros Organics (Geel, Belgium) and Sigma-Aldrich (St Louis, MO, USA) and were used without further purification. All solvents were distilled before use. The microelemental analysis for CHNS-O was carried out using a Carlo Erba 1108 Elemental Analyzer (Milan, Italy). The infrared spectrum (IR) of the product (KBr pellets) was recorded using a Perkin Elmer Spectrum GX spectrophotometer (Perkin Elmer, Waltham, MA, USA) in the range of 400–4,000 cm^−1^. Nuclear Magnetic Resonance (^1^H and ^13^C) experiments were performed on a Bruker 600 MHz instrument using DMSO-d_6_ as the solvent. TGA was performed under flowing nitrogen at a heating rate of 10 °C min^−1^ using a Mettler Thermogravimetric Analyzer (Mettler-Toledo, Poslfach, Switzerland).

### 3.2. Preparation of C-5-bromo-2-hydroxyphenylcalix[4]-2-methylresorcinarene *(**I**)*

Concentrated hydrochloric acid (7 mL) was added into a round-bottom flask containing a solution of 5-bromo-2-hydroxybenzaldehyde (0.01 mol, 2.01 g) in absolute ethanol (60 mL). The mixture was stirred for 30 minutes and a solution of 2-methylresorcinol (0.01 mol, 1.24 g) in absolute ethanol (20 mL) was added. The mixture was refluxed for 24 hours at 80 °C. The yellow precipitate formed was collected by filtration, washed with distilled water and acetone several times and dried under vacuum. Yield (76%); FTIR (KBr, cm^−1^): 3340 (OH), 1433 (C=C), 1211 (C-O), 609 (C-Br); ^1^H-NMR (600 MHz; precipitate, DMSO-d_6_) δ_H_: 1.94 (6H, s, Ar-CH_3_), 2.11 (6H, s, Ar-CH_3_), 5.37 (2H, s, Ar-CH), 5.91 (4H, s, Ar-CH), 6.10 (2H, s, Ar-CH), 6.34 (4H, s, Ar-H), 6.38 (4H, d, J = 8.0, OH-Ar-CH), 6.77 (4H, s, OH), 6.83 (4H, d, *J* = 8.0, Br-Ar-CH), 7.48 (4H, s, OH), 8.94 (4H, s, OH); ^13^C-NMR (150 MHz; DMSO-d_6_) δ_C_: 10.4 (2 ° CH_3_), 10.6 (2 ° CH_3_), 36.8 (4 ° CH), 110.5 (3 ° Ar-CH_3_), 110.8 (Ar-CH_3_), 112.1 (4 ° Ar-H), 116.3 (4 ° Ar-Br), 120.5 (4 ° Ar-CH), 123.8 (4 ° Ar-CH), 125.6 (2 ° Ar-H), 127.5 (2 ° Ar-H), 128.7 (4 ° Ar-H), 132.9 (4 ° Ar-CH), 133.2 (4 ° Ar-H), 150.9 (4 ° Ar--OH), 151.2 (4 ° Ar-OH), 153.8 (4 ° Ar-OH). ^1^H-NMR (600 MHz; crystallized, DMSO-d_6_) δ_H_: 1.95 (6H, s, Ar-CH_3_), 2.12 (6H, s, Ar-CH_3_), 2.73 (30H, s, 10 ° CH_3_, DMF), 2.89 (30H, s, 10 ° CH_3_, DMF), 5.38 (2H, s, Ar-CH), 5.91 (4H, s, Ar-CH), 6.10 (2H, s, Ar-CH), 6.34 (4H, s, Ar-H), 6.38 (4H, d, *J* = 8.0, OH-Ar-CH), 6.77 (4H, s, OH), 6.83 (4H, d, *J* = 8.0, Br-Ar-CH), 7.48 (4H, s, OH), 7.95 (10H, s, 10 x COH, DMF), 8.95 (4H, s, OH). (Crystal). Anal. Calcd for (molecular formula): C = 54.75 and H = 3.61 Found: C, 54.22 and H, 3.59. Suitable yellowish crystals for X-ray investigation were obtained by recrystallization from DMF but changed to a powder after a few hours exposure to air. Coating the fresh crystals with Princeton-H oil allowed the X-ray experiment to be conducted for at least 10 hours.

### 3.3. X-ray Crystallography

Single-crystal X-ray experiment was performed on Bruker D-QUEST diffractometer (Bruker, AXS Inc., Madison, WI, USA) using graphite-monochromated Mo-Kα radiation (λ = 0.71073 Å). Intensity data was measured at 100(2) K by the ω-scan. Accurate cell parameters and orientation matrix were determined by the least-squares fit of 25 reflections. Intensity data was collected for Lorentz and polarization effects. Empirical absorption correction was carried out using multi-scan. The structure was solved by direct methods and least-squares refinement of the structure was performed by the SHELXL-97 program [20]. All the non-hydrogen atoms were refined anisotropically. The hydrogen atoms were placed in calculated positions, allowing them to ride on their parent C atom with U_iso_(H) = 1.2U_eq_ except the hydrogen atoms of the water solvates were located from Fourier maps and refined isotropically. A summary of the data collections and details of the structure refinement is given in Table 5. Crystallographic data for the structural determination has been deposited with the Cambridge Crystallographic Data Center, CCDC No 959177. This information may be obtained free of charge at http://www.ccdccam.ac.uk/const/retrieving.html or from the Cambridge Crystallographic Centre (CCDC), 12 Union Road, Cambridge CB2, 1EZ, UK (Fax: +44(0)-1223-336033; E-Mail: deposit@ccdc.cam.ac.uk.

**Table 5:** Crystallographic data and structural refinement of C-5-bromo-2-hydroxy phenylcalix[4]-2-methylresorcinarene.

Crystal parameters	Data/values	
CCDC deposition number	959177	
Empirical formula	C_80_H_108_Br_4_N_8_O_24_	
Moiety formula	C_56_H_44_Br_4_O_12_, 8(C_3_H_7_NO), 4(H_2_O)	
Formula weight	1885.38	
Temperature	100(2) K	
Wavelength λ	0.71073 Å	
Crystal system	Triclinic	
Space group	Pī	
Unit cell dimensions	a = 15.9592(16) Å	α = 68.656(3)°
	b = 16.9417(17) Å	β = 85.689(3)°
	c = 17.0974(17) Å	γ = 81.631(3)°
Volume	4258.6(7) Å3	
Z	2	
D_cal_ (Mg/m^3^)	1.470	
Absorption coefficient	1.969 mm−1	
F(000)	1952	
Crystal dimension (mm)	0.42 × 0.37 × 0.24	
T_min_/T_max_	0.4918, 0.6494	
Reflections measured	130536	
Ranges/indices (h,k,l)	−19, 19; −20, 20; −21, 21	
θ limits (º)	2.8 to 26.0°	
Unique reflections	16701	
Observed reflections (I>2σ(I))	11455	
Parameters	1101	
Goodness of fit on F^2^	1.13	
R_1_, wR_2_ (I≥2σ(I))	0.0661, 0.1652	
R_1_,wR_2_ indices (all data)	0.1107, 0.1997	
Largest diff. peak and hole	2.669 and −0.977 e.Å−3	

### 3.4. Antioxidant Test

A stock solution of DPPH was prepared by dissolving DPPH (0.4 g) in methanol (1 L) and the solution was kept in the dark at 4 °C. A stock solution of the C-5-bromo-2-hydroxyphenylcalix[4]-2-methylresorcinarene (**I**) was prepared at 10 mg/5 mL in DMSO. A volume of 100 µL from the stock solution of the compound was added to 1 mL of DPPH. The mixture was shaken well and kept in the dark at room temperature for 2 hours. The absorbance of the mixture was measured at 517 nm using a spectrophotometer. The percentage of inhibition of radical scavenging ability was calculated as:

Inhibition % = [(A_DPPH_ – A_Sample_)/(A_DPPH_)] × 100 = [(1.012 − 0.1523)/1.012] × 100 = 84.95%.

### 3.5. Antibacterial Activity

Antibacterial activity was determined by the disc diffusion method [21] followed by minimum inhibitory concentration (MIC) and minimum bactericidal concentration (MBC) tests against two Gramnegative and three Gram-positive bacteria. A stock solution at 25 mg/mL was prepared by dissolving C-5-bromo-2-hydroxyphenylcalix[4]-2-methylresorcinarene (**I**, 25 mg) in dimethyl sulfoxide (DMSO, 1 mL). Two fold-dilution of the stock solution was prepared to produce test solutions at 12.5, 6.250, 3.125 and 1.563 mg/mL.

Test bacteria were grown in Mueller-Hinton broth (MHB) and bacterial suspensions standardized to 2 × 10^8^ cells/mL were used to prepare bacterial lawn on Mueller-Hinton agar (MHA). After allowing the disc to dry for 5 minutes, five sterile Whatman No: 1 filter paper discs (6 mm in diameter) were placed on the bacterial lawn. Test solution (5 μL) at different concentrations was applied to the paper discs using a micropipettor. Vancomycin or chloramphenicol was used as positive controls. DMSO was used as a negative or solvent control. Plates were incubated at 37 °C for 24 hours of incubation and the diameter of inhibition zone was measured (Figure 6).

The minimum inhibition concentration (MIC) determination was carried out on compound **I** using the broth dilution method in 96 well microplate according to Andrews [22]. The minimum bactericidal concentration (MBC) was determined by subculturing the broth in wells with test compound that showed no growth on nutrient agar (NA). The concentration that showed no growth on the NA plates was determined and the MBC.

Selectivity index is determined using the CC_50_ from the cytotoxicity evaluation and the MIC and calculated as SI = CC_50_/MIC.

### 3.6. Cytotoxicity Evaluation

The cytotoxicity of the compound was first determined on uninfected Vero cells (African monkey *Cercophiteus aetiops* kidney cells) with dilutions ranging from 5 mg/mL to 0.039 mg/mL in Dulbecco’s Modified Eagle’s Medium (DMEM, Flowlab, North Ride, Australia). Cytotoxicity was determined using the MTT assay [23]. The CC_50_ value that is the compound concentration that kills 50% of the cell population was determined by the optical density of solubilized formazan. The percentage of growth inhibition was calculated using the following formula:

% Cell viability = 100 × (Abs/Ac)

where Abs = Absorbance value of test compound and Ac = Absorbance value of control (cells only).

### 3.7. Antiviral Activity

The plaque reduction assay was performed to study the presence of antiviral activity of compound **I**. HSV-1 stock was prepared and the viral titre of the stock was determined as 5.7 × 10^8^ pfu/mL. The concentration of compound **I** used in this assay was set based on the concentration that allows growth of 70% or more Vero cells. This is to ensure that cell death is not due to the toxicity of the test compound which will affect the accuracy of the test results. In this study, the first test concentration of C-5-bromo-2-hydroxophenylcalix[4]-2-methylresorcinarene (**I**) is 0.35 mg/mL, which is below the CC_50_ value and continues with two fold serial dilutions until 0.011 mg/mL. Virus was infected to 80% confluent cells and incubated for 48 hours for plaque formation. Plaques were stained with crystal violet and the numbers of plaques were counted. The EC_50_ value was determined as the concentration that inhibited plaque formation by 50% of the untreated cells [24,25].

## 4. Conclusions

C-5-bromo-2-hydroxyphenylcalix[4]-2-methylresorcinarene (**I**) was successfully synthesized by the cyclocondensation of 5-bromo-2-hydroxybenzaldehyde and 2-methylresorcinol in the presence of concentrated HCl. The X-ray structure was in good agreement with the NMR data and the calix molecule adopted a chair *C*_2h_ conformation. Compound **I** showed good anti-HSV-1 and antioxidant activity at non-cytotoxic concentrations, but weak antibacterial activity.

## Supplementary Material

Supplementary material can be accessed at: http://www.mdpi.com/1420-3049/18/11/13369/s1.
